# Annual acknowledgement of manuscript reviewers

**DOI:** 10.1186/s13033-016-0051-0

**Published:** 2016-03-09

**Authors:** Harry Minas

**Affiliations:** University of Melbourne, Melbourne, Australia

## Abstract

With the proliferation of journals and the ever-increasing demands on the time and expertise of reviewers it has become increasingly difficult for editors to get high quality and timely reviews. On behalf of authors as well as the Journal the editors of *International Journal of Mental Health Systems* would like to thank all our reviewers who have made an indispensible contribution to the journal in Volume 9 (2015).

**Helal Uddin Ahmed**

Bangladesh

**Qais Alemi**

USA

**Gjumrakch Aliev**

USA

**Peter Allebeck**

Sweden

**Noor Amawi**

USA

**Gregory Armstrong**

Australia

**Joseph Asare**

Ghana

**Cecilia Aslund**

Sweden

**Michelle Banfield**

Australia

**Concepción Barrio**

USA

**Peter Bartlett**

UK

**Carolina Borges**

Brazil

**Lisa Boss**

USA

**David Burke**

Australia

**Meena Cabral de Mello**

Switzerland

**José Miguel Caldas de Almeida**

Portugal

**Maureen Canavan**

USA

**Önver Cetrez**

Sweden

**Sudipto Chatterjee**

India

**Rie Chiba**

Japan

**Alex Cohen**

UK

**Judith Cohen**

USA

**Erminia Colucci**

Australia

**Annette Crisanti**

USA

**Wendy Cross**

Australia

**Joachim Diederich**

Australia

**Elizabeth Dinapoli**

USA

**Michael Dunne**

Australia

**Vivian Dzokoto**

USA

**Julian Eaton**

UK

**Alberto Ferguson**

Colombia

**Deede Gammon**

Norway

**Eshetu Girma**

Ethiopia

**Daniela Goncalves Bradley**

UK

**Enrique Gracia**

Spain

**Margaret Grigg**

Australia

**Oye Gureje**

Nigeria

**Teresa Hall**

Australia

**Nutmeg Hallett**

UK

**Laura Hart**

Australia

**Edvard Hauff**

Norway

**Ashraf Hosseini**

Australia

**Asma Humayun**

Pakistan

**Abdallah Ibrahim**

Ghana

**Petru Ifteni**

Romania

**Afzal Javed**

UK

**Michael Jones**

Australia

**Anthony Jorm**

Australia

**Ritsuko Kakuma**

Australia

**Harry Kennedy**

Ireland

**Susan Kidd**

Australia

**John Kjøbli**

Norway

**Manami Kodaka**

Japan

**Brandon Kohrt**

USA

**Janice Kopinak**

Canada

**Irene Kretchy**

Ghana

**Nhan La**

Australia

**Tero Laiho**

Finland

**Yik-Wa Law**

Hong Kong

**Lars Lien**

Norway

**Elizabeth Lin**

Canada

**Lijun Liu**

China

**Weishu Liu**

China

**Anthony Mannarino**

USA

**Niall McCrae**

UK

**Peter McGovern**

Switzerland

**Graham Meadows**

Australia

**Glenn Melvin**

Australia

**Damian Mohan**

Ireland

**Mervyn Morris**

UK

**Laura K. Murray**

USA

**David Ndetei**

Kenya

**Sudan Neupane**

Norway

**Thi Mai Hien Nguyen**

Viet Nam

**Angela Nickerson**

Australia

**Anne Marie O’Keefe**

USA

**Kwaku Oppong Asante**

Ghana

**Claire O’Reilly**

Australia

**Jong-Ik Park**

 Democratic People’s Republic of Korea

**Sidandi Paul**

Botswana

**Veikko Pelto-Piri**

Sweden

**Inge Petersen**

South Africa

**Michael Phillips**

China

**Asheq Rahman**

Australia

**Carla Rash**

USA

**Paula Reavey**

UK

**Rusty Reeves**

USA

**Siobhan Reilly**

UK

**Heather Robinson**

UK

**Dyann Ross**

Australia

**Alison Salloum**

USA

**Dyaa Saymah**

UK

**Nasima Selim**

Bangladesh

**Akihiro Shiina**

Japan

**Siham Sikander**

Pakistan

**Peter Silverstone**

Canada

**William Sledge**

USA

**Dayanandan Somasundaram**

Sri Lanka

**Robert Stewart**

Malawi

**Rupert Suckling**

UK

**Long Sun**

China

**Carlos Suso-Ribera**

Spain

**Nora Sveaass**

Norway

**Theonie Tacticos**

Australia

**Hwang Tae-Yeon**

Republic Of Korea

**Leslie Terry**

Australia

**Murali Thyloth**

India

**Jane Topolovec-Vranic**

USA

**Athena Tsai**

Taiwan

**Sonsoles Valdivia-Salas**

Spain

**Mark Van Ommeren**

Switzerland

**Bregje Van Spijker**

Australia

**Ruth Benedicte Veenhuizen**

Netherlands

**Tua Vinther-Jensen**

Denmark

**Wei Chun Wang**

Australia

**Xingmin Wang**

China

**Laura White**

USA

**Harvey Whiteford**

Australia

**Mirkuzie Woldie**

Ethiopia

**Alexandra Wright**

Australia

**Fu Yang**

China

